# Prevalence and Predictors of Colposcopic-Histopathologically Confirmed Cervical Intraepithelial Neoplasia in HIV-Infected Women in India

**DOI:** 10.1371/journal.pone.0008634

**Published:** 2010-01-08

**Authors:** Vikrant V. Sahasrabuddhe, Ramesh A. Bhosale, Smita N. Joshi, Anita N. Kavatkar, Chandraprabha A. Nagwanshi, Rohini S. Kelkar, Cathy A. Jenkins, Bryan E. Shepherd, Seema Sahay, Arun R. Risbud, Sten H. Vermund, Sanjay M. Mehendale

**Affiliations:** 1 Institute for Global Health, Vanderbilt University, Nashville, Tennessee, United States of America; 2 Department of Obstetrics and Gynecology, Byramjee Jeejeebhoy Medical College, Pune, India; 3 Department of Epidemiology, National AIDS Research Institute, Pune, India; 4 Department of Pathology, Byramjee Jeejeebhoy Medical College, Pune, India; 5 Department of Microbiology, Tata Memorial Centre, Mumbai, India; 6 Department of Biostatistics, Vanderbilt University, Nashville, Tennessee, United States of America; 7 Department of Social and Behavioral Science, National AIDS Research Institute, Pune, India; 8 Department of Microbiology, National AIDS Research Institute, Pune, India; University of Cape Town, South Africa

## Abstract

**Background:**

Prevalence estimates of cervical intraepithelial neoplasia (CIN) among HIV-infected women in India have been based on cervical cytology, which may have underestimated true disease burden. We sought to better establish prevalence estimates and evaluate risk factors of CIN among HIV-infected women in Pune, India using colposcopy and histopathology as diagnostic tools.

**Methodology:**

Previously unscreened, non-pregnant HIV-infected women underwent cervical cancer screening evaluation including standardized diagnostic colposcopy by a gynecologist. Histopathologic confirmation was conducted among consenting women with clinical suspicion of CIN. The prevalence of CIN was evaluated by a composite diagnosis based on colposcopy and histopathology results. Multivariable ordinal logistic regression analysis was conducted to determine independent predictors of increasing severity of CIN.

**Results:**

The median age of the n = 303 enrolled HIV-infected women was 30 years (interquartile range, 27–34). A majority of the participants were widowed or separated (187/303, 61.7%), more than one-third (114/302, 37.7%) were not educated beyond primary school, and nearly two-thirds (196/301, 64.7%) had a family per capita income of <1,000 Indian Rupees (∼US$22) per month. Cervical high-risk HPV-DNA was detected in 41.7% (124/297) of participants. The composite colposcopic-histopathologic diagnoses revealed no evidence of CIN in 220 out of 303 (72.6%) women, CIN1 in 33/303 (10.9%), CIN2 in 31/303 (10.2%), CIN3 in 18/303 (5.9%) and 1 (0.3%) woman was diagnosed with ICC. Thus, over a quarter of the participants [83/303: 27.7% (95% CI: 22.7–33.1)] had ≥CIN1 lesions and a sixth [50/303: 16.5% (95% CI: 12.2–21.9)] had evidence of advanced (≥CIN2) neoplastic disease. The independent predictors of increasing severity of CIN as revealed by a proportional odds model using multivariable ordinal logistic regression included (i) currently receiving antiretroviral therapy [adjusted odds ratios (aOR): 2.24 (1.17, 4.26), p = 0.01] and (ii) presence of cervical high-risk HPV-DNA [aOR: 1.93 (1.13, 3.28), p = 0.02].

**Conclusions:**

HIV-infected women in Pune, India have a substantial burden of cervical precancerous lesions, which may progress to invasive cervical cancer unless appropriately detected and treated. Increased attention should focus on recognizing and addressing this entirely preventable cancer among HIV-infected women, especially in the context of increasing longevity due to antiretroviral therapy.

## Introduction

Women living with human immunodeficiency virus (HIV) infection have a higher risk of human papillomavirus (HPV)-associated cervical intraepithelial neoplasia (CIN) as compared to HIV-uninfected women. [1], [2] India has some of the highest case burdens of both HIV/AIDS (estimated 2.4 million individuals, including 1 million women) and cervical cancer (estimated 130,000 new cases and 74,000 deaths annually) of any single nation.[3]–[5] The life span of HIV-infected women in India is increasing due to improved access to affordable antiretroviral therapy (ART). [6] Before the introduction of ART, the lack of cervical cancer prevention services probably had little influence on the life expectancy of HIV-infected women due to high competing mortality associated with other opportunistic infections. [7], [8] As HIV-infected women continue to live longer with ART support, albeit in a moderately immunosuppressed state, they may be at increased risk for CIN and invasive cervical cancer.[1], [7]

Prior studies have reported an increased risk of cervical intraepithelial neoplasia (CIN) in HIV-infected women in India and other developing regions, but have almost entirely relied on the detection of precancerous lesions using cervical cytology for documenting prevalence.[9]–[12] However, with its low to moderate sensitivity for detecting CIN, cytology may have underestimated the true prevalence of CIN in HIV-infected women.[13], [14] Furthermore, even in clinical practice, an abnormality on cytology needs confirmation by diagnostic colposcopy and further by histopathology (if indicated) to reveal true disease status and plan appropriate treatment.[15], [16] It is important to assess the extent of the disease burden through well-designed prevalence studies that may inform targeting of scant resources for prevention intervention activities. Unfortunately, no prior studies have reported colposcopic-histopathologically confirmed prevalence of CIN in HIV-infected women from India. We undertook a descriptive epidemiology study in Pune, India to determine the prevalence and predictors of colposcopic-histopathologically confirmed CIN among HIV-infected women.

## Methods

### Study Setting and Participants

We developed an outpatient colposcopy clinic in a tertiary care hospital in Pune, India. Study participation was offered to consecutive HIV-infected women in a public-sector ART center in the hospital premises. Participants were also recruited through outreach efforts among self-help groups of HIV-infected women in Pune city. Eligibility criteria included having documented serologic evidence of HIV infection, negative urine pregnancy test, absence of debilitating illness that may preclude a pelvic examination, no prior history of screening or treatment for cervical neoplasia, and no prior hysterectomy. Women with syndromic evidence of sexually transmitted infections (STI) were initially managed as per World Health Organization (WHO) guidelines before continuing enrollment.[17] Participants were recruited regardless of their CD4+ cell counts or current ART status.

### Study Procedures

**Figure 1:**
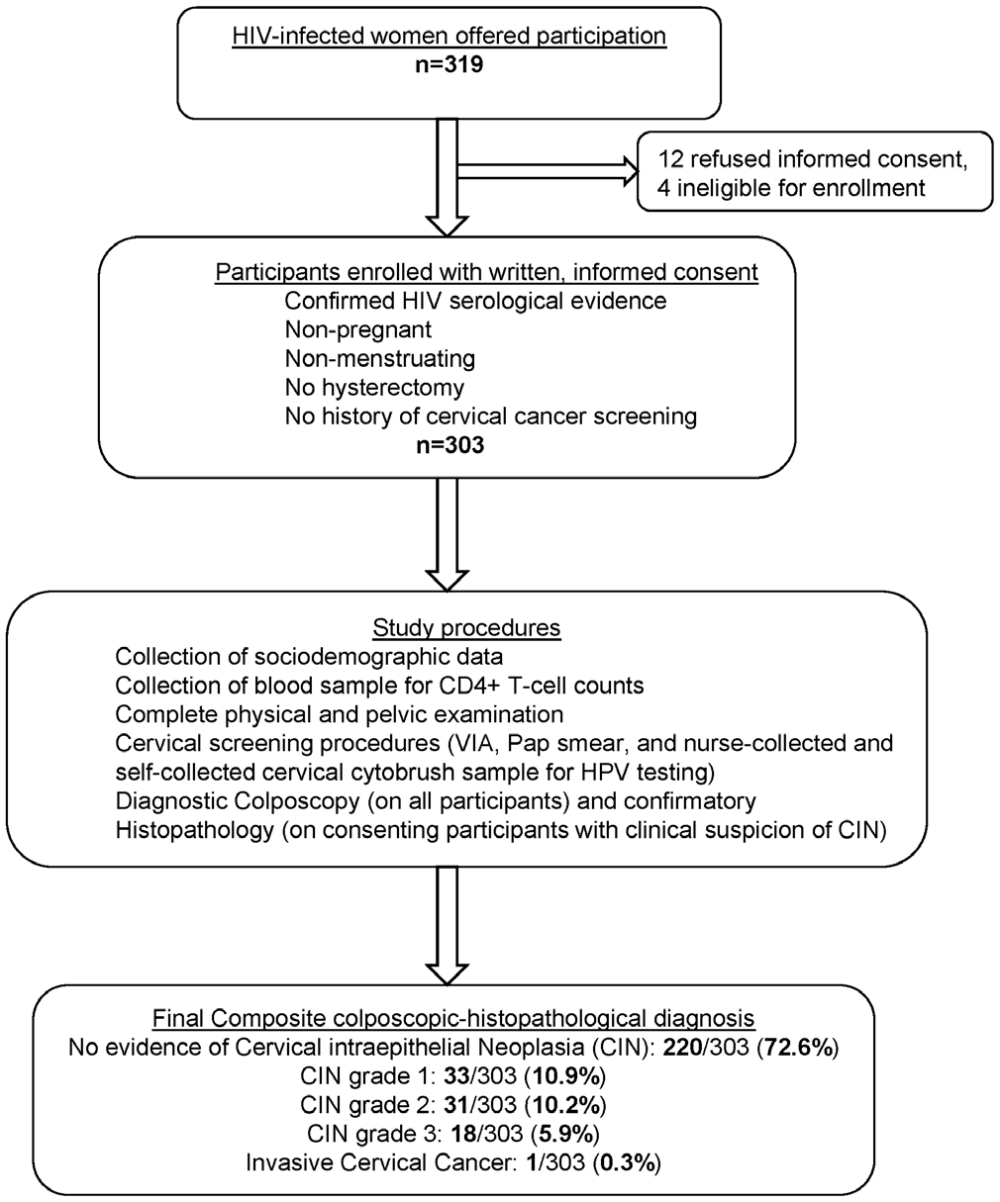
Study enrollment, procedures, and main outcomes. The flow diagram shows the number of HIV-infected women who were offered participation, those enrolled in the study, list of study procedures, and final diagnosis through the composite colposcopic-histopathological assessment.

After explanation of study procedures and written informed consent, a structured questionnaire was administered to interview the participants and collect their sociodemographic information as well as sexual and reproductive history (sexual behaviors, obstetric history, menstrual history, past history of STI) and medical history relevant to HIV/AIDS and cervical cancer. A blood sample was obtained for CD4+ T-cell counts estimation [FACSCount™ flow cytometry, Becton, Dickinson and Company, Franklin Lakes, NJ, USA]. All enrolled women underwent a complete physical, pelvic, and colposcopic examination. Trained nurses collected endocervical samples that were tested for presence of high-risk HPV-DNA by the Digene Hybrid Capture 2™ (HC-2) assay [Qiagen, Inc., Gaithersburg, MD, USA] in a certified laboratory. [18] Nurses also collected samples for Pap smears and performed visual inspection with acetic acid (VIA) exam. All participants provided a self-collected vaginal swab for HPV testing. (Results of the accuracy of screening tests will be reported in a separate manuscript). A standardized non-invasive colposcopy examination was performed on ***all*** participants by gynecologists who recorded colposcopic diagnosis using the Reid's scoring index.[19] Invasive confirmatory procedures [including cervical punch biopsies, endocervical curettage (ECC), and loop electrosurgical excision procedures (LEEP)] were advised and performed on consenting participants with clinical evidence of cervical abnormalities. Histopathology samples from these procedures were analyzed independently by two experienced pathologists who reported diagnosis by consensus. The final diagnosis was based on (i) histopathology results for women in whom invasive procedures were performed and (ii) diagnostic colposcopy results in women who had no clinical indication for undergoing invasive procedures, or in whom histopathology was unavailable or inconclusive. Results were reported as per the Richart CIN staging system in five categories of increasing disease severity: normal/no neoplastic abnormalities, CIN1, CIN2, CIN3, and invasive cervical cancer (ICC). [20] The study procedures and results are summarized in the flow diagram. (Figure 1)

### Statistical Methods

The statistical analyses were performed using R statistical software (Version 2.9.1; http://www.r-project.org). The five-level diagnostic categories were collapsed into a four-level ordinal outcome variable of increasing neoplastic severity (i.e. no CIN, CIN1, CIN2, and ≥CIN3) for statistical analyses. We evaluated the predictors of increasing grade (severity) of CIN (on the ordered outcome variable: no CIN, CIN1, CIN2, and ≥CIN3) using bivariate (unadjusted) and multivariable ordinal logistic regression analyses. Odds ratios (OR), 95% confidence intervals (CI) and two-tailed p-values were calculated.[21] Ordinal logistic regression assumes proportional odds – the association between predictor variables and the odds of severe disease is constant regardless of the CIN cut-off used to classify disease severity. This assumption was visually inspected [22], and appeared reasonable. The multivariable model included covariates found to be statistically significant (p<0.05) on unadjusted analysis as well as those deemed *a priori* to be most biologically salient to CIN incidence and progression. To avoid over-fitting, the multivariable model was limited to 10 covariates based on frequencies within the ordered outcome variable.[23] For all models, continuous covariates were first included in the model using restricted cubic splines to avoid linearity assumptions. If the non-linear portions did not prove to be significant, the model was refit assuming linearity. We assumed that missing values occurred at random given the other covariates and used multiple imputation to derive predictions for missing values.[22] The extent of completeness of data for various predictor variables is represented in Table 1.

**Table 1:** Distribution of sociodemographic variables as per final disease outcomes (confirmed by colposcopic histopathological diagnoses) among HIV-infected women in Pune, India.

*Covariates*		*Overall*	*Normal*	*CIN1*	*CIN2*	*CIN3 and ICC*
Overall		100% (303)	72.6% (220)	10.9% (33)	10.2% (31)	6.3%(19)
Age		n = 303				
	Median (IQR) (years)	30 (27, 34)	30 (27,33.3)	32(28,36)	29(25,31.5)	31(28,40)
Education		n = 302				
	No/some primary education	38% (114)	34% (75)	39% (13)	52% (16)	53% (10)
	High school or above	62% (188)	66% (144)	61% (20)	48% (15)	47% (9)
Marital status		n = 303				
	Cohabiting with husband/spouse	38% (116)	37% (82)	24% (8)	55% (17)	47% (9)
	widowed/separated	62% (187)	63% (138)	76% (25)	45% (14)	53% (10)
Family income		n = 301				
	<INR 1,000∧/month/person	65% (196)	66% (144)	64% (21)	61% (19)	63% (12)
	≥INR 1,000∧/month/person	35% (105)	34% (74)	36% (12)	39% (12)	37% (7)
Occupation		n = 302				
	Employed/Professional	66% (200)	68% (148)	61% (20)	61% (19)	68% (13)
	Unemployed/Housewife	34% (102)	32% (71)	39% (13)	39% (12)	32% (6)
Age at first sex		n = 298				
	Median (IQR) (years)	18 (15, 19)	18 (16,19)	18 (15,19)	17 (15,18)	16 (15.3,17)
Age at menarche		n = 299				
	Median (IQR) (years)	13 (13, 14)	13(13,14)	14 (13,15)	13 (12,14)	13.5 (13,14)
Lifetime sexual partners		n = 297				
	≥2 partners	13% (37)	10% (21)	27% (9)	20% (6)	6% (1)
	1 partner	87% (260)	90% (196)	73% (24)	80% (24)	94% (16)
HIV status of partner		n = 300				
	HIV-infected spouse	31% (92)	29% (64)	18% (6)	48% (15)	37% (7)
	No HIV-infected spouse	69% (208)	71% (153)	82% (27)	52% (16)	63% (12)
Condom Use frequency		n = 118				
	Not using/inconsistent user	45% (53)	47% (40)	43% (3)	35% (6)	50% (4)
	Consistent/always user	55% (65 )	53% (46)	57% (4)	65% (11)	50% (4)
History of past STI		n = 303				
	Yes, history of past STI	32% (96)	31% (68)	33% (11)	39% (12)	26% (5)
	No history of past STI	68% (207)	69% (152)	67% (22)	61% (19)	74% (14)
Use of tobacco products		n = 303				
	Current user/used in past	40% (122)	41% (91)	33% (11)	35% (11)	47% (9)
	Never used	60% (181)	59% (129)	67% (22)	65% (20)	53% (10)
Used hormonal contraceptive ≥1 year		n = 303				
	Used ≥1 year	8% (25)	8% (17)	9% (3)	10% (3)	11% (2)
	Not used/used <1 yr	92% (278)	92% (203)	91% (30)	90% (28)	89% (17)
Number of births		n = 299				
	Median (IQR)	2 (1,3)	2 (1,3)	2 (1,3)	2 (1,3)	3 (2,3)
Months since HIV detection		n = 151				
	Median (IQR)	23 (5,39)	23 (6, 38)	31 (13, 45)	9 (3, 33)	25 (8, 37)
Presence of gynecological symptoms		n = 303				
	Symptoms present currently	90 (30%)	31% (68)	27% (9)	26% (8)	26% (5)
	No symptoms present currently	213 (70%)	69% (152)	73% (24)	74% (23)	74% (14)
Body Mass Index		n = 303				
	Median (IQR)	19.4(17.8,21.3)	19.4 (17.8,21.2)	19.2 (18,21.5)	19.7 (17.8,20.7)	19.7 (17.1,22.4)
CD4+ T-cell count		n = 293				
	Median (IQR) (/µL)	343 (241,497)	355 (260, 497)	343 (252, 500)	264 (183, 510)	327 (237,400)
WHO Stage		n = 303				
	Stage III/IV	14% (41)	13% (29)	12% (4)	13% (4)	21% (4)
	Stage I/II	87% (262)	87% (191)	88% (29)	87% (27)	79% (15)
ART status		n = 271				
	Currently receiving ART	26% (70)	21% (42)	38% (11)	25% (7)	67% (10)
	ART-naïve	74% (201)	79% (157)	62% (18)	75% (21)	33% (5)
Presence of cervical high-risk HPV DNA		n = 297				
	high-risk HPV-DNA positive	42% (124)	37% (80)	52% (17)	50% (15)	67% (12)
	high-risk HPV DNA negative	58% (173)	63% (136)	48% (16)	50% (15)	33% (6)

Abbreviations: IQR: interquartile ratio (25th percentile and 75th percentile), INR: Indian Rupees; BMI: Body Mass Index; ART: Antiretroviral therapy; STI: Sexually transmitted infection; WHO: World Health Organization.

∧ The exchange rate at the time of the study was approximately 45 INR (Indian Rupees) per US$ such that Indian Rupees 1,000 are the equivalent of US$ 22.

### Ethics Statement

The study protocol was approved by the institutional review board of Vanderbilt University, and the ethics committees of the National AIDS Research Institute (NARI) and Byramjee Jeejeebhoy Medical College (BJMC). Scientific and administrative approvals for this Indo-U.S. collaborative research study were also received from the Scientific Advisory Committee of NARI and the Indian Health Ministry Screening Committee, with endorsement from the Indian National AIDS Control Organization. All participants gave written informed consent.

## Results

### Sociodemographic Profile

Participation was offered to 319 HIV-infected women between October 2006 and September 2007, of whom two each (0.6% each) were excluded due to pregnancy or prior hysterectomy. Of 315 eligible persons, 303 (96%) enrolled with informed consent. The median age was 30 years (interquartile range, IQR: 27–34). A majority of the participants were widowed or separated (187/303, 61.7%) as opposed to cohabiting with their husbands or spouses (116/303, 38.3%). More than one-third of the participants (114/302, 37.7%) were not educated beyond primary school and nearly two-thirds (196/301, 64.7%) had a family per capita income of less than 1,000 Indian Rupees (approximately US$22 at the time) per month. A large majority of the participants (260/297, 87.5%) reported only one lifetime sexual partner. The median reported age at first sexual intercourse was 18 years (IQR: 15–19), median age of menarche was 13 years (IQR: 13–14) and the median number of births per woman was 2 (IQR: 1–3). The median CD4+ T-cell count was 343/µL (IQR: 244–495) and a large majority (262/303; 86.5%) presented with WHO Clinical Stage I/II of HIV/AIDS. About one quarter of women (70/271, 25.8%) were on ART at the time of enrollment; however, detailed data were not available on ART regimen or their duration of treatment. Cervical high-risk HPV-DNA (by the HC-2 assay) was detected in nurse-collected cervical swabs in about two-fifths [41.7% (124/297)] of the participants.

### Cervical Disease Prevalence

Colposcopy was performed on all participants and revealed no evidence of CIN in 223 women, CIN1 in 17 women, CIN2 in 27 women, and CIN3 in 36 women. No cases of ICC were detected on colposcopy. Invasive diagnostic procedures for histopathology were indicated in a total of 73/303 (24.1%) women undergoing colposcopy. This included 63 women with the protocol defined threshold of offering histopathological confirmatory procedures for evidence of ≥CIN2 on colposcopy [36 women with CIN3 and 27 women with CIN2], as well as in 10 women for whom the clinician deemed that histopathology was necessary [including 4 women who had an “unsatisfactory” colposcopic examination and 6 women having colposcopic CIN1 lesions with suspicion of endocervical extension of lesions that prompted need for histopathological confirmation]. (Table 2)

**Table 2:** Distribution of the histopathologic diagnoses stratified by colposcopy results among HIV-infected women in Pune, India.

		Colposcopy results					
		No CIN	CIN1	CIN2	CIN3	ICC	Total
**Histopathologic diagnoses**							
	No Histopathology done	219	11	7	7	0	244
	Histopathology inconclusive	0	0	2	0	0	2
	No CIN	0	0	0	1	0	1
	CIN1	2*	4∧	9	7	0	22
	CIN2	2*	1∧	6	13	0	22
	CIN3	0	1∧	3	7	0	11
	ICC	0	0	0	1	0	1
		223	17	27	36	0	303

* = these 4 participants were indicated histopathological confirmation due to an “unsatisfactory” colposcopic examination result.

∧ = these 6 participants had CIN1 lesions on colposcopy but the clinician suspected endocervical lesions prompting histopathological confirmation.

Of the 73 women for whom histopathology was recommended, 59 actually underwent the procedures, which included 29 out of 36 with CIN3 on colposcopy, 20 out of 27 with CIN2 on colposcopy, and all 10 with clinical indications for undergoing histopathology. Fourteen participants (7 with CIN2 and 7 with CIN3) did not consent to undergo histopathological confirmatory procedures. (The most common cited reason was the necessity to consult with their family members or spouses before undergoing an invasive procedure. In spite of efforts by study staff to recall them for a follow-up visit to undergo the procedures, these 14 participants did not return.) Histopathological results were reported as “inconclusive” in 2 participants (having CIN2 lesions on colposcopy) for whom there were no possibility of recall for repeat procedures. Thus, the colposcopy results served as the final diagnosis in 246/303 (81.2%) women, which included the 14 participants who refused the invasive procedures and 2 participants with inconclusive histopathology results. Histopathology results, available in 57/303 (18.8%) participants revealed 1 woman with no evidence of CIN, 22 women with CIN1, 22 women with CIN2, 11 women with CIN3, and a singular case of ICC (a participant who had CIN3 impression on colposcopy). (Table 2)

The final composite colposcopic-histopathologic diagnoses revealed no abnormality (no evidence of CIN) in 220 out of 303 (72.6%) women. CIN1 was reported in 33 (10.9%) women, CIN2 in 31 (10.2%) women, CIN3 in 18 (5.9%) women, while 1 (0.3%) woman was diagnosed with ICC. (Table 3) Thus, more than 1 in 4 women [83/303: 27.7% (95% CI: 22.7–33.1)] had colposcopic-histopathological evidence of ≥CIN1 lesions and 1 in 6 women [50/303: 16.5% (95% CI: 12.2–21.9)] had evidence of advanced (≥CIN2) neoplastic disease.

**Table 3:** Distribution of the “composite” colposcopic histopathological diagnoses by the confirmatory diagnostic procedure (colposcopy or histopathology) among HIV-infected women in Pune, India.

		Result based on Histopathology diagnoses	Result based on Colposcopy results
Final “Composite” diagnosis			
	No CIN = 220	1	219
	CIN1 = 33	22	11
	CIN2 = 31	22	9
	CIN3 = 18	11	7
	ICC = 1	1	0
	Total = 303	57	246

### Predictors of Increasing Severity of CIN

In unadjusted (bivariate) analysis, participants with greater odds of more severe CIN included those currently receiving ART, those with cervical high-risk HPV-DNA, those with two or more lifetime sexual partners (marginally significant) and those educated only up to the primary school level. (Table 4) On multivariable ordinal logistic regression analysis, the independent predictors of having greater odds of more severe CIN were (i) currently receiving ART [adjusted odds ratios (aOR): 2.24 (95% CI: 1.17, 4.26), p = 0.01] as compared to being ART-naïve, and (ii) having presence of cervical high-risk HPV-DNA [aOR: 1.93 (1.13, 3.28), p = 0.01] as opposed to being cervical high-risk HPV-DNA-negative. (Table 4) HIV-infected women with primary school education or lower [aOR: 1.85 (0.97, 3.51), p = 0.06] and those with two or more lifetime sexual partners [aOR: 1.80 (0.91, 3.59), p = 0.09] also had greater odds of more severe CIN, but these associations were only marginally statistically significant. (Table 4)

**Table 4:** Unadjusted (bivariate) and multivariable ordinal logistic regression analysis of predictors of disease prevalence for ordinal outcome in three categories of increasing disease severity (No CIN, CIN1, CIN2, ≥CIN3) confirmed by composite colposcopic-histopathological diagnosis among HIV-infected women in Pune, India.

	Unadjusted (bivariate) ordinal logistic regression analysis		Multivariable ordinal logistic regression analysis*	
	OR (95% CI)	p-value	Adjusted OR (95% CI)	p-value
Unit (1-year) increase in age (e.g., 28 vs. 27 years)	1.03 (0.99, 1.08)	0.15	1.01 (0.96, 1.06)	0.71
No/some primary education (vs. ≥high school)	**1.76 (1.06, 2.91)**	**0.03**	1.85 (0.98, 3.51)	0.06
Cohabiting with husband/spouse (vs. ≥widowed/separated)	1.28 (0.77, 2.13)	0.34	*Not included*	**–**
Family income <INR 1,000/month/person (vs. ≥INR 1,000)∧	0.86 (0.51, 1.45)	0.58	0.85 (0.48, 1.51)	0.59
Employed/Professional (vs. unemployed/housewife)	0.83 (0.49, 1.39)	0.48	*Not included*	–
Unit (1-yr) increase in age at first sex (e.g.,18 vs. 17 yrs)	0.94 (0.86, 1.02)	0.14	0.98 (0.89, 1.09)	0.72
Unit (1-yr) increase in age at menarche (e.g.,13 vs. 12 yrs)	1.03 (0.85, 1.24)	0.80	*Not included*	–
≥2 lifetime sexual partners (vs. single lifetime partner)	1.91 (0.98, 3.73)	0.06	1.80 (0.91, 3.59)	0.09
HIV-infected spouse (vs. no HIV-infected spouse)	1.33 (0.78, 2.26)	0.30	*Not included*	**–**
No/inconsistent condom use (vs. consistent use)	0.81 (0.36, 1.82)	0.61	*Not included*	–
History of presence of STI (vs. no STI)	1.12 (0.66, 1.89)	0.68	*Not included*	–
Ever used tobacco products (vs. never users)	0.88 (0.53, 1.48)	0.64	*Not included*	–
Hormonal contraceptive ≥1 year (vs. not used/used <1 yr)	1.28 (0.54, 3.04)	0.57	*Not included*	–
Unit increase in number of births (e.g., 3 vs. 2 births)	1.06 (0.87, 1.30)	0.55	0.99 (0.79, 1.25)	0.93
Unit incr. in months since HIV detection (e.g., 13 vs. 12)	0.94 (0.78, 1.13)	0.48	*Not included*	–
Presence of gynecological symptoms (vs. no symptoms)	0.81 (0.46, 1.41)	0.45	*Not included*	–
Unit increase in BMI (e.g., 20 vs. 19)	0.99 (0.91, 1.08)	0.82	*Not included*	–
100 unit increase in CD4+ count (/µL) (e.g., 350 vs. 250)	0.92 (0.81, 1.04)	0.18	0.96 (0.84, 1.10)	0.55
WHO Stage III/IV (vs. WHO Stage I/II)	1.17 (0.57, 2.40)	0.67	1.07 (0.50, 2.32)	0.86
Currently receiving ART (vs. ART-naïve)	**2.48 (1.40, 4.40)**	**<0.01**	**2.24 (1.17, 4.26)**	**0.01**
Presence of cervical high-risk HPV DNA (vs. no high-risk HPV DNA)	**2.06 (1.24, 3.42)**	**<0.01**	**1.93 (1.13, 3.28)**	**0.02**

Abbreviations: OR: Odds ratio; CI: Confidence intervals; INR: Indian Rupees; BMI: Body Mass Index; ART: Antiretroviral therapy; STI: Sexually transmitted infection; WHO: World Health Organization.

* The number of covariates that could be included in the multivariable regression model was dependent on the number of event outcomes for that analysis to avoid over-fitting of the models. Hence only 10 covariates were included in the multivariable model. Covariates were deemed scientifically important and selected *a priori* along with covariates that were significant in unadjusted (bivariate) analysis.

∧ The exchange rate at the time of the study was approximately 45 INR (Indian Rupees) per US$ such that Indian Rupees 1,000 are the equivalent of US$ 22.

## Discussion

Our study is the first report from India on CIN disease prevalence in HIV-infected women confirmed by a composite colposcopic-histopathological assessment. Previous studies have relied on the use of cytology to report prevalence of CIN in HIV-infected women.[9]–[12]. The cytology-derived prevalence estimates in these studies have varied according to the population being sampled, ranging from 6.3% (voluntary counseling and testing clinics, n = 287),[11] 14% (HIV and gynecology clinics, n = 75),[12] 19.2% (women attending STI clinics, n = 100),[9] to 30.7% (commercial sex workers, n = 137),[9]. However, cervical cytology (Pap smear) has only moderate sensitivity in detecting CIN and may miss many lesions.[13], [14] Besides, ‘true’ CIN disease estimation is only done by standardized diagnostic confirmatory colposcopy followed by histopathological confirmation as may be indicated clinically. [15], [16] All participants in our study received a standardized diagnostic colposcopic examination. Additional invasive confirmatory diagnostic procedures (biopsy, LEEP, ECC) were restricted only for consenting women with clinical evidence of CIN, as per internationally recommended clinical management guidelines.[24] This approach avoided unnecessary invasive diagnostic procedures yet increased the accuracy of CIN diagnosis more than from diagnostic colposcopy alone.[24], [25] Thus, our study design represents the best effort for maximizing the precision of clinical-pathological prevalence estimates while balancing ethical concerns by avoiding unnecessary invasive procedures. The participant recruitment in this study was undertaken regardless of the baseline CD4+ cell counts or presence of symptoms of cervical cancer or HIV/AIDS. This, and the fact that none of the participants had been previously screened or treated for cervical neoplasia, has limited the selection bias in our study and increased the generalizability and representativeness of our findings for HIV-infected women in India.

A limitation of our data was that we were not able to extract complete data on duration of ART or type of ART regimens. While national guidelines in India suggest that ART be initiated if nadir CD4+ cell counts fall under 200/µL or if there is presence of AIDS-defining illness, our data only includes the CD4+ cell counts at the time of enrollment (as opposed to nadir CD4+ cell counts). In our multivariable analyses, currently receiving ART for HIV/AIDS was an independent predictor of CIN, even after controlling for current CD4+ T-cell counts and stage of HIV disease. While evidence on the impact of ART on the natural history of CIN in HIV-infected women is limited, most studies have pointed that immune reconstitution due to ART has minimal or no impact on the progression of CIN. [26], [27] Thus, as HIV-infected women in India and elsewhere in the developing nations start living longer on ART in a moderately immunosuppressed state, they may continue to be at risk for slowly progressing cervical neoplasia associated with persistent HPV infection unless preventive interventions are instituted.

Our study also confirms the high prevalence (41.7%) of high-risk HPV-DNA in this population, as well as the presence of HPV as an independent predictor of CIN, as observed in multiple studies among HIV-infected women worldwide.[28]–[30] Although HPV-DNA testing currently remains unavailable or unaffordable for most women in India and other developing countries, there is a significant global push toward the introduction of low-cost, rapid HPV testing in developing countries that may allow better diagnostic options for CIN, especially for high-risk HIV-infected women.[31], [32]

Our study has several limitations. Colposcopy and histopathology results are by nature rater-dependent and subjective interpretations.[33]–[38] We tried to address this limitation by standardizing the interpretation of colposcopic diagnoses (using Reid's scoring index) and incorporating quality assurance of colposcopic images by a senior experienced gynecologist (RB). The histopathology reports were read by two independent pathologists (AK, CN) who initially analyzed each histopathology slide and then reconciled differences and reported results by consensus. Yet the possibility of misclassification of lesions cannot be eliminated completely, especially considering the transitory nature of HPV infection and low grade (CIN1) lesions in HIV-infected women.[39] Also, some experts have recommended use of invasive cervical biopsy for histopathology even from normal looking areas of the cervix during colposcopy for improved diagnostic accuracy rather than using selective directed histopathology of colposcopically abnormal appearing lesions alone.[40], [41] While we performed ECC whenever possible in situations where colposcopy did not reveal abnormalities, our study protocol emphasized the avoidance of unnecessary invasive procedures to prevent risk of iatrogenic infection and untoward blood loss in our cohort of HIV-infected women. This may have led us to overly rely on colposcopic assessment as opposed to histopathological assessment in a majority of our participants. Yet, performing invasive procedures from normal appearing parts of the cervix is controversial, and there is clearly a role for improving disease ascertainment through non-invasive novel screening and diagnostic tools.[32], [42] Finally, the cross-sectional nature of this study allows the possibility for uncontrolled confounding and also precludes any attempt for deriving causality. Indeed, this underscores the need for conducting well-designed prospective cohort studies to study natural history of cervical neoplasia among HIV-infected women in developing country settings. We have initiated prospective evaluation of this cohort and established similar study cohorts in both urban and rural sites in other high-prevalence settings in India that may also improve the generalizability of our findings.

The need for cervical cancer screening services for this population, as evidenced by the high prevalence of advanced cervical neoplasia in this study (≥CIN1: 27.7% and ≥CIN2: 16.6%), is enormous. Yet the population coverage of cervical cancer prevention programs in India and most developing countries is largely inadequate.[43], [44] While limitations in initiating and sustaining services for cervical cancer prevention services are key concerns, the coverage is also poor since a majority of women in these settings hardly access clinical care, let alone preventive care.[45] Fortunately, HIV/AIDS care and treatment programs represent a potential window of opportunity for providing preventive clinical interventions to avert a cervical cancer epidemic nested within the HIV pandemic. We believe that providing cost-effective, life-saving cervical cancer prevention services linked to HIV care (much like screening and treatment for tuberculosis and other opportunistic infections) must be a global imperative for HIV-infected women who have an elevated risk for this entirely preventable malignancy.[46], [47] In the context of the global economic downturn and the resulting challenges in public health allocations, integrated sexual and reproductive health care programs such as HIV/AIDS care and cervical cancer prevention may allow efficient utilization of resources for improving health care access for those most vulnerable to these eminently preventable diseases.
